# Commencement Exercises, Veterinary Department, Detroit College of Medicine

**Published:** 1895-10

**Authors:** 


### COMMENCEMENT EXERCISES, VETERINARY DEPARTMENT, DETROIT COLLEGE OF MEDICINE.

THE fourth annual commencement exercises were held on the evening of April 3, in Prismatic Hall. The graduating class were favored with an Address by Senator Palmer, who strongly discountenanced many of the operations of fashion of the day. The class address was delivered by Herbert F. Palmer; subject, "Three Decades in American History." The degrees were conferred upon the following graduates: H. Buchanan, H. A. King, H. V. Smith, of Detroit, Mich.; A. Deadman, of Alpena, Mich.; J. F. Deadman, of Sault Ste. Marie, Mich.; H. F. Palmer, of Napoleon, Mich.; and A. J. Savage, of Litchfield, Ill. The graduating exercises were completed by a meeting of the Veterinary Medical Society, when certificates of membership were granted to the graduating class. The Veterinary Department now has twenty-two graduates and thirteen junior matriculants.

